# Hydrogen Sulfide Attenuates Hydrogen Peroxide-Induced Injury in Human Lung Epithelial A549 Cells

**DOI:** 10.3390/ijms20163975

**Published:** 2019-08-15

**Authors:** Mingqi Wang, Xinyu Cao, Chang Luan, Zhengqiang Li

**Affiliations:** Key Laboratory for Molecular Enzymology and Engineering of Ministry of Education, College of Life Sciences, Jilin University, Changchun 130012, China

**Keywords:** Hydrogen sulfide, hydrogen peroxide, reactive oxygen species, endoplasmic reticulum stress, lung injury

## Abstract

Lung tissues are frequently exposed to a hyperoxia environment, which leads to oxidative stress injuries. Hydrogen sulfide (H_2_S) is widely implicated in physiological and pathological processes and its antioxidant effect has attracted much attention. Therefore, in this study, we used hydrogen peroxide (H_2_O_2_) as an oxidative damage model to investigate the protective mechanism of H_2_S in lung injury. Cell death induced by H_2_O_2_ treatment could be significantly attenuated by the pre-treatment of H_2_S, resulting in a decrease in the Bax/Bcl-2 ratio and the inhibition of caspase-3 activity in human lung epithelial cell line A549 cells. Additionally, the results showed that H_2_S decreased reactive oxygen species (ROS), as well as neutralized the damaging effects of H_2_O_2_ in mitochondria energy-producing and cell metabolism. Pre-treatment of H_2_S also decreased H_2_O_2_-induced suppression of endogenous H_2_S production enzymes, cystathionine-beta-synthase (CBS), cystathionine-gamma-lyase (CSE), and 3-mercapto-pyruvate sulfurtransferase (MPST). Furthermore, the administration of H_2_S attenuated [Ca^2+^] overload and endoplasmic reticulum (ER) stress through the mitogen-activated protein kinase (MAPK) signaling pathway. Therefore, H_2_S might be a potential therapeutic agent for reducing ROS and ER stress-associated apoptosis against H_2_O_2_-induced lung injury.

## 1. Introduction

Hydrogen sulfide (H_2_S) is a poisonous, flammable gas, with the smell of rotten eggs, and is simply regarded as an environmental pollutant [1]. Recently, H_2_S has been considered the third signaling gasotransmitter, accompanying nitric oxide and carbon monoxide, due to its multiple functions in physiological and pathological processes [2,3]. Studies have shown that H_2_S participates in cardiovascular remodeling, cell proliferation, migration and invasion, oxidative stress, and inflammation [4,5,6]. Endogenous H_2_S is mainly produced from L-cysteine via reactions catalyzed by three enzymes: cystathionine-beta-synthase (CBS), cystathionine-gamma-lyase (CSE), and 3-mercapto-pyruvate sulfurtransferase (MPST) [7,8]. Recent reports have shown that a multitude of H_2_S-releasing small-molecule drugs, such as H_2_S-hybrid nonsteroidal anti-inflammatory drugs (HS-NSAIDS), showed a significant reduction of gastrointestinal damage compared to the parent NSAIDS [9,10].

The lung is the most important respiratory organ involved in gas exchange that is frequently in contact with the ambient air, including nitrogen dioxide, sulfur dioxide, ozone, cigarette smoke, and diesel exhaust [11,12]. Exposure to a hyperoxia environment increases the intracellular production of reactive oxygen species (ROS) [13]. Meanwhile, a large amount of superoxide anions caused by viral infection, drugs, or surgery also results in oxidative stress injuries [14,15,16]. ROS induces structural and functional abnormalities in the mitochondrial electron transport chain, and the imbalance between antioxidants and oxidants can cause cell injury and even death [17,18]. Therefore, ROS is the basis of many lung diseases.

The endoplasmic reticulum (ER) is an organelle responsible for the synthesis, folding, assembly, and modification of proteins [19]. In pathological conditions, ER dysfunction and calcium dyshomeostasis lead to an excessive accumulation of unfolded or misfolded proteins, which induces ER stress [20]. A growing number of studies have suggested that ER stress plays critical roles in many physiological and pathological processes, including allergy and inflammation, Alzheimer’s disease, cardiovascular disease, and obesity [21,22,23,24]. ER stress is also a common cause of lung diseases, indicating that a misfolded protein may be an important unifying mechanism in pulmonary fibrosis and even lung cancer [25].

Our previous study found that sodium hydrosulfide (NaHS), as the exogenous H_2_S donor, regulated the cell proliferation and angiogenesis of A549 cells. Despite its function in tumor growth, the effects of H_2_S on ER stress in the human lung epithelial cell line A549 remain to be elucidated. In the present study, hydrogen peroxide (H_2_O_2_) was chosen as the model of lung injury in vitro. We investigated whether H_2_S protected A549 cells against oxidation-induced ER stress. In this paper, we present evidence that ER stress contributed to H_2_O_2_-induced apoptosis in human lung epithelial cells via the MAPK signaling pathway. Our research provides a novel treatment of oxidative-related diseases with H_2_S.

## 2. Results

### 2.1. Protective Effect of H_2_S on Human Lung Epithelial Cells against H_2_O_2_-Induced Apoptosis

**Figure 1:**
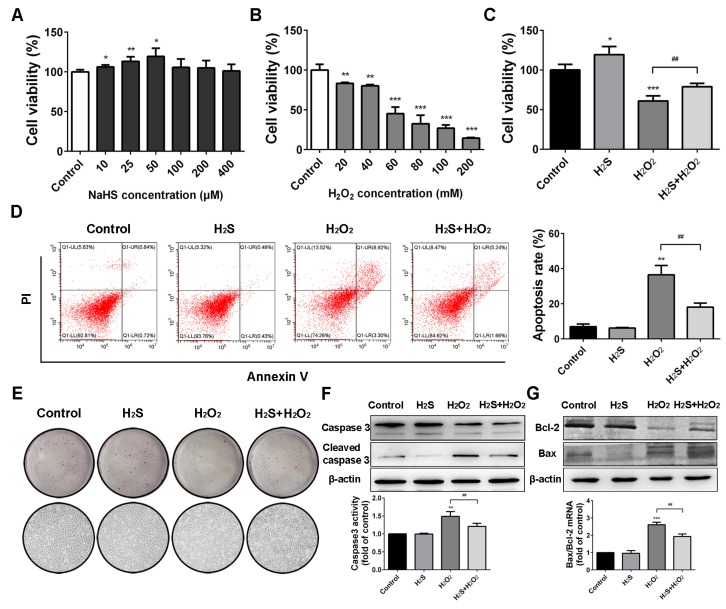
Hydrogen sulfide (H_2_S) reduced hydrogen peroxide (H_2_O_2_)-induced injury in A549 cells. (**A**) Cell viability assay. After incubation with sodium hydrosulfide (NaHS) at 0 (control), 10, 25, 50, 100, 200, and 400 μM for 12 h, A549 cell viability was tested via a 3-(4, 5-dimethylthiazol-2-yl)-2, 5-diphenyltetrazolium bromide (MTT) assay. (**B**) MTT assay. A549 cells were treated with 20, 40, 60, 80, 100, and 200 mM H_2_O_2_ for 12 h and then subjected to an MTT assay. (**C**) MTT assay. A549 cells treated with serum-free medium, 50 μM H_2_S, and 60 mM H_2_O_2_ were the control group, H_2_S group, and H_2_O_2_ group, respectively. A549 cells pre-treated with H_2_S (50 μM) for 2 h and then subjected to H_2_O_2_ (60 mM) for 10 h were the protection group (H_2_S+H_2_O_2_). (**D**) Flow cytometric cell apoptosis assay. Histograms depict proportions of total apoptotic cells. (**E**) Cell colony formation and microscopic morphology (200×). (**F**) The protein expression of caspase 3 was measured using western blot analysis and the caspase 3 activity was measured using a caspase 3 assay kit. (**G**) The mRNA expression and protein expression of Bax and Bcl-2 were detected using quantitative real-time PCR (qRT-PCR) and western blot analysis. The experiments were repeated at least three times. The results are presented as the mean ± SD. (* *p* < 0.05, ** *p* < 0.01, *** *p* < 0.001 vs. corresponding control group; ## *p* < 0.01 H_2_O_2_ group vs. H_2_S + H_2_O_2_ group).

As shown in Figure 1A, different concentrations of NaHS were added to the culture medium of human lung epithelial cells for 12 h. Cell activities increased most obviously in 50 μM NaHS treatment. A549 cells were then incubated with different concentrations of H_2_O_2_ for 12 h, and the half maximal inhibitory concentration (IC_50_) was 60.25 mM (Figure 1B). Therefore, the concentration of 60 mM was chosen for the model of H_2_O_2_-induced lung injury. The cell state in the H_2_S + H_2_O_2_ group was better than that in the H_2_O_2_ group under an optical microscope (Figure 1E). To further determine whether H_2_S had a protective effect on H_2_O_2_-induced apoptosis, A549 cells were pre-treated with H_2_S for 2 h and then subjected to H_2_O_2_. As shown in Figure 1C, H_2_S obviously reduced H_2_O_2_ cytotoxicity. The flow cytometric analysis confirmed that the cell apoptosis rate in the H_2_S pre-treatment group was significantly decreased compared with that in the H_2_O_2_ group (Figure 1D). A colony formation assay indicated that the A549 cells in the H_2_O_2_ group were barely forming clones. With pre-treatment of H_2_S, A549 cells could form a few clones (Figure 1E). The expression of apoptosis genes was further examined. Compared to the untreated cells, A549 cells expressed more cleaved-caspase 3 when treated with H_2_O_2_. Meanwhile, the activity of caspase 3 was decreased in the protection group compared with the injury group (Figure 1F). The ratio of Bax and Bcl-2 mRNA expression was markedly decreased when pre-treated with H_2_S compared to that in the H_2_O_2_ group. Western blot analysis was in agreement with the quantitative real-time PCR (qRT-PCR) results, showing that the protein expression of Bax was up-regulated and Bcl-2 was down-regulated in the H_2_O_2_ group. H_2_S pre-treatment could decrease Bax expression and increase Bcl-2 expression (Figure 1G). These results indicated that H_2_S reduced H_2_O_2_-induced injury in lung epithelial cells.

### 2.2. Protection Effect of H_2_S on Reactive Oxygen Species Injury

**Figure 2:**
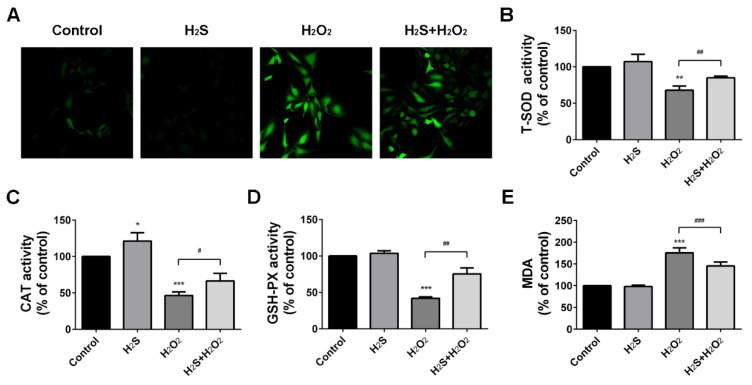
Hydrogen sulfide (H_2_S) protected A549 cells against hydrogen peroxide (H_2_O_2_)-induced oxidative stress. (**A**) Intracellular superoxide anion production was detected with the dihydroethidium 2′,7′-dichloroflurescein-diacetate (DCFH-DA) and observed by a laser scanning confocal microscope (400×). (**B**–**D**). The activities of superoxide dismutase (SOD), catalase (CAT), and glutathione peroxidase (GSH-PX) were measured. (**E**) The malondialdehyde (MDA) production was measured. The experiments were repeated at least three times. The results are presented as the mean ± SD. (* *p* < 0.05, ** *p* < 0.01, *** *p* < 0.001 vs. corresponding control group; # *p* < 0.05, ## *p* < 0.01, ### *p* < 0.001 H_2_O_2_ group vs. H_2_S + H_2_O_2_ group).

Upon H_2_O_2_ treatment, a massive production of intracellular reactive oxygen species (ROS) was observed. However, ROS production could be suppressed by pre-treatment of H_2_S (Figure 2A). Superoxide dismutase (SOD) and glutathione peroxidase (GSH-PX) are the main oxygen free radical scavengers [26]. Catalase (CAT) is involved in peroxide breakdown and malondialdehyde (MDA) is one of the final products of polyunsaturated acid peroxidation [27]. Therefore, we decided to test the activities of SOD, GSH-PX, and CAT, and the MDA production in A549 cells. Our data showed that SOD, GSH-PX, and CAT activities were decreased in the H_2_O_2_ group compared to the control group. However, these phenomena were completely reversed by H_2_S pre-treatment (Figure 2B–D). As shown in Figure 2E, H_2_O_2_ obviously increased MDA production, while H_2_S pre-treatment efficiently lowered the content of MDA. These findings suggested that H_2_S could attenuate H_2_O_2_-induced oxidative stress in A549 cells.

### 2.3. H_2_O_2_ Suppressed Endogenous H_2_S Production in A549 Cells

**Figure 3:**
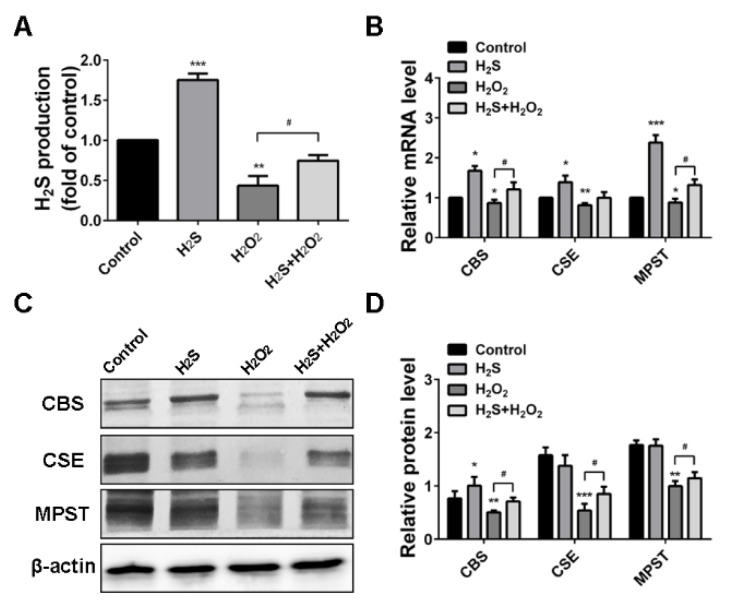
Hydrogen peroxide (H_2_O_2_) suppressed endogenous hydrogen sulfide (H_2_S) production and H_2_S-producing enzymes. (**A**) H_2_S production tested using a methylene blue assay. (**B**) Quantitative real-time PCR (qRT-PCR) assay results for cystathionine-beta-synthase (CBS), cystathionine-gamma-lyase (CSE), and 3-mercapto-pyruvate sulfurtransferase (MPST) mRNA expression levels. (**C**) Western blot analysis of CBS, CSE, and MPST protein expression levels. (**D**) Quantitative analysis of CBS, CSE, and MPST band intensities. The experiments were repeated at least three times. The results are presented as the mean ± SD. (* *p* < 0.05, ** *p* < 0.01, *** *p* < 0.001 vs. corresponding control group; # *p* < 0.05, ## *p* < 0.01 H_2_O_2_ group vs. H_2_S + H_2_O_2_ group).

Endogenous H_2_S production was measured to further determine the effects of oxidative stress induced by H_2_O_2_. The results showed that the rates of H_2_S production were significantly increased with H_2_S treatment, while blocked by H_2_O_2_ treatment (Figure 3A). Inhibitory functions of H_2_O_2_ were confirmed by qRT-PCR analysis, which demonstrated that H_2_O_2_ suppressed H_2_S-producing enzymes CBS, CSE, and MPST expression (Figure 3B). Western blot analysis showed the same trend with the mRNA expression. H_2_O_2_ resulted in significant CBS, CSE, and MPST expression inhibition. However, pre-treatment of H_2_S attenuated the reduction effect induced by H_2_O_2_ (Figure 3C,D). Therefore, we preliminarily determined that H_2_O_2_-induced oxidative injury was associated with endogenous H_2_S-producing enzymes.

### 2.4. Effect of H_2_S on H_2_O_2_ Injury A549 Cells in Mitochondrial Membrane Potential (Δψ) and Energy Metabolism

**Figure 4:**
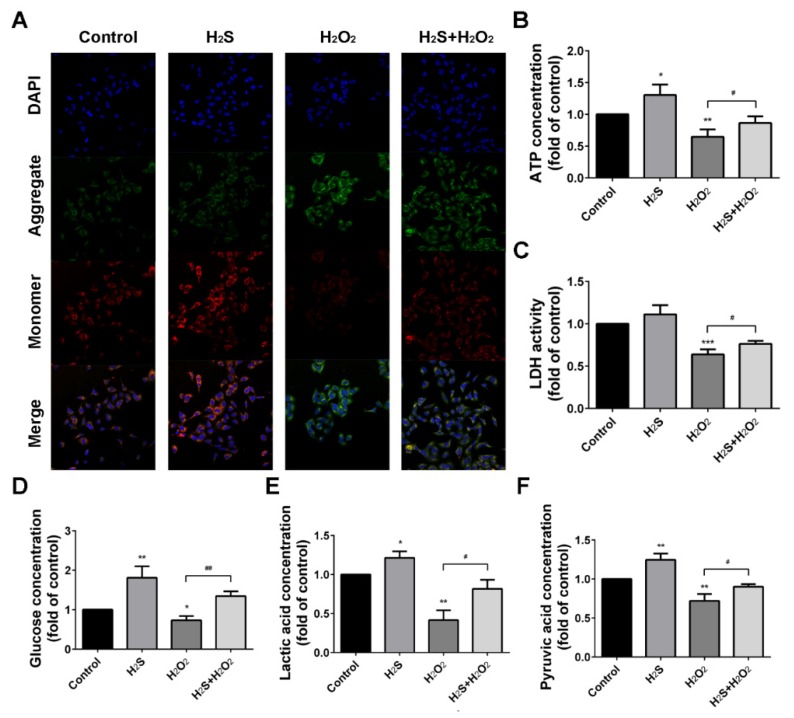
Hydrogen sulfide (H_2_S) restores hydrogen peroxide (H_2_O_2_)-induced reduction of mitochondrial membrane potential (Δψ) and energy metabolism. (**A**) The Δψ was determined by lipophilic cationic probe JC-1 with a laser scanning confocal microscope (400×). Red signal indicated JC-1 in the mitochondrial matrix and green signal indicated JC-1 in cytosol. (**B**) ATP production (μmol/g protein) and (**C**) the enzymatic activity of lactate dehydrogenase (LDH) in A549 cells were measured. (**D**–**F**) Glucose consumption (μmol/mg protein), lactic acid production (mmol/g protein), and pyruvate uptake (μmol/mg protein) were measured with assay kits. The experiments were repeated at least three times. The results are presented as the mean ± SD. (* *p* < 0.05, ** *p* < 0.01 vs. corresponding control group; # *p* < 0.05, ## *p* < 0.01 H_2_O_2_ group vs. H_2_S + H_2_O_2_ group).

Mitochondrial function is highly susceptible to ROS injury, and the change in mitochondrial membrane potential (Δψ) is the sign of damage [28]. We investigated whether H_2_S regulated Δψ change induced by H_2_O_2_. Mitochondria in the control group exhibited high Δψ, which showed red fluorescence. However, mitochondria showed less intense red fluorescence, but more intense green fluorescence, with H_2_O_2_ exposure. H_2_S pre-treatment could improve the red fluorescence intensity, which illustrated that H_2_S might prevent the loss of Δψ (Figure 4A). H_2_O_2_-induced mitochondrial function injury directly resulted in the reduction of the ATP output, while H_2_S could increase ATP production (Figure 4B). With H_2_O_2_ treatment, the enzymatic activity of lactate dehydrogenase (LDH) was decreased compared to the control group (Figure 4C). Meanwhile, our results showed that H_2_S accelerated the metabolic process, resulting in an increase of glucose consumption, lactic acid production, and pyruvate uptake (Figure 4D,F). These findings indicated that H_2_O_2_ decreased the mitochondrial membrane potential, as well as energy metabolism progress.

### 2.5. Effect of H_2_S on ROS-Induced Intracellular [Ca^2+^] and Endoplasmic Reticulum Stress

**Figure 5:**
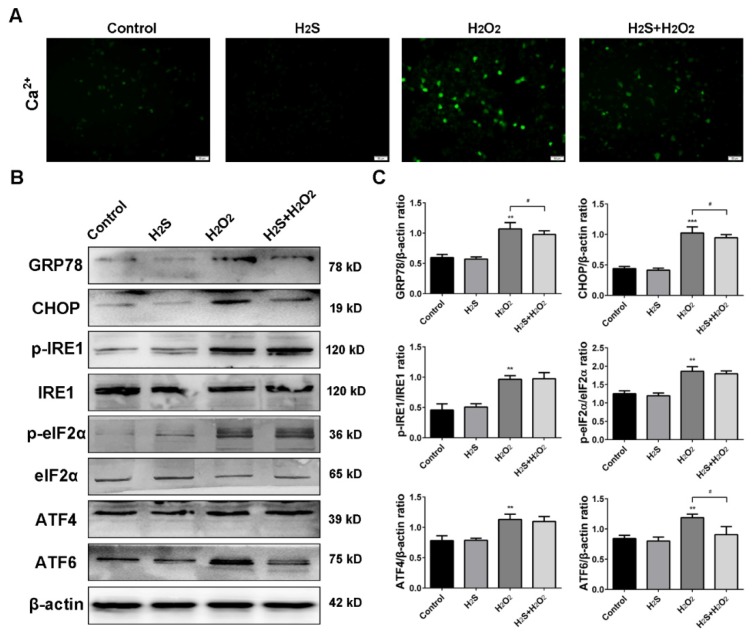
Hydrogen sulfide (H_2_S) attenuates [Ca^2+^] and endoplasmic reticulum (ER) stress induced by reactive oxygen species (ROS). (**A**) The calcium ion levels were detected with fluorescence probe Fluo-3, AM by a fluorescence microscope (200×). (**B**) Western blot analysis of GRP78, CHOP, phospho-IRE1, phospho-eIF2α, ATF4, and ATF6 upon different treatment. (**C**) Bar graphs show quantification of the protein levels. The experiments were repeated at least three times. The results are presented as the mean ± SD. (* *p* < 0.05, ** *p* < 0.01, *** *p* < 0.001 vs. corresponding control group; # *p* < 0.05 H_2_O_2_ group vs. H_2_S + H_2_O_2_ group).

The ER lumen is the main storage of [Ca^2+^], and ER dysfunction promotes the calcium output [20]. We first detected intracellular [Ca^2+^] with the Fluo-3, AM fluorescence probe, and the results showed that H_2_S limited the [Ca^2+^] overload under H_2_O_2_ stress (Figure 5A). GRP78 and CHOP, the two main ER stress markers, were measured by western blot analysis. As shown in Figure 5B, H_2_O_2_ injury contributed to the overexpression of GRP78 and CHOP, but H_2_S pre-treatment blocked these increases. We further investigated the effect of H_2_S on the ER stress pathway. H_2_O_2_ stimulated the phosphorylation expression of IRE1 and eIF2α, and then up-regulated ATF4 and ATF6. However, H_2_S significantly decreased the level of ATF6, but had a slight effect on p-IRE1, p-eIF2α, and ATF4. Therefore, we preliminarily determined that H_2_O_2_ caused calcium overload via ER stress.

### 2.6. Effect of H_2_S on the Mitogen-Activated Protein Kinase (MAPK) Signaling Pathway in H_2_O_2_-Treated A549 Cells

**Figure 6:**
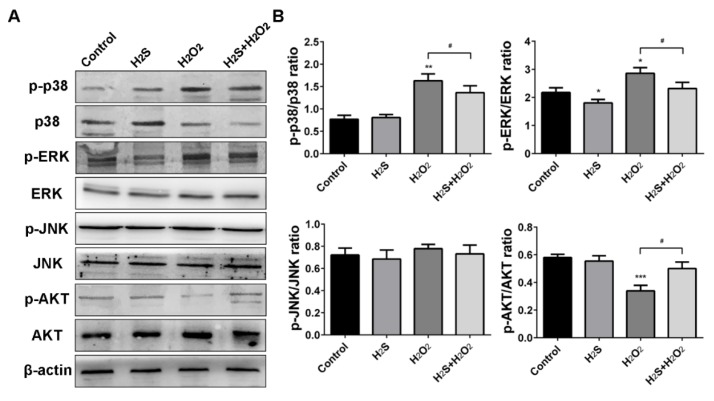
Hydrogen sulfide (H_2_S) mediates hydrogen peroxide (H_2_O_2_)-induced mitogen-activated protein kinase (MAPK) activation in A549 cells. (**A**) The protein expressions of phospho-p38, phospho-ERK 1/2, phospho-JNK, and phospho-AKT were analyzed by western blot analysis. (**B**) Bar graphs shows quantification of the protein levels. The experiments were repeated at least three times. The results are presented as the mean ± SD. (* *p* < 0.05, ** *p* < 0.01, *** *p* < 0.001 vs. corresponding control group; # *p* < 0.05 H_2_O_2_ group vs. H_2_S + H_2_O_2_ group).

ROS play a crucial part in cells via regulation of the MAPK signaling pathway [29]. Herein, phosphorylated levels of p38, ERK, JNK, and AKT were examined via western blot analysis to investigate whether H_2_O_2_ was involved in MAPK pathway activation. The results showed that the phosphorylation of p38 and ERK was up-regulated and the phosphorylation of AKT was suppressed by H_2_O_2_ treatment, compared with that in the control group. However, H_2_S pre-treatment decreased the p-p38 and p-ERK, but had little effect on p-JNK expression (Figure 6A,B). Therefore, the results suggested that H_2_S mediated H_2_O_2_-induced MAPK activation in A549 cells.

## 3. Discussion

Recently, a growing number of researches indicate that H_2_S participates in the regulation of various physiological and pathological processes in the human body [5,30,31]. Our research found that 10–50 μM NaHS promoted cell proliferation. Therefore, the cyto-protective effect of H_2_S could only be achieved at a low level of NaHS. The lung is susceptible to a hyperoxia environment, and excessive ROS production can damage the physiological functions of lung tissue, such as epithelial function, endothelial cells, and airway smooth muscle [32]. Therefore, preventing oxidative stress has become an important target for lung diseases. In this study, we investigated whether exogenous H_2_S attenuated ROS-induced injury in human lung epithelial A549 cells. The results showed that H_2_S protected A549 cells from H_2_O_2_-induced apoptosis, maintained the redox balance, and defended the oxidative stress. H_2_S pre-treatment also preserved mitochondrial membrane potential, which was essential for ATP production and energy metabolism. Moreover, H_2_S attenuated intracellular [Ca^2+^] and ER stress induced by ROS.

H_2_O_2_ is widely used to model the oxidative stress of mammalian cells. The cell morphology, survival rate, and expression of apoptosis-associated proteins were detected to ensure that the oxidative injury model was successfully established for the subsequent experiments. The results showed that the A549 cell survival rate was 53.72 ± 5.31% with stimulation of 60 mM H_2_O_2_, while the cell activity was increased by 17.75 ± 4.69% for the H_2_O_2_ group with 50 μM H_2_S pre-treatment. Moreover, lactate dehydrogenase, which is located in the cytoplasm of normal cells, but is released into the cell culture medium when cells are injured, is a sensitive marker of cell damage. H_2_O_2_ significantly increased LDH release (Figure S1), which demonstrated that the model was successfully constricted.

High levels of ROS could lead to an imbalance of the cellular redox state and oxidative stress, as well as induce cell apoptosis [33]. Therefore, we chose to measure intracellular reactive oxygen species, glutathione, superoxide dismutase, and malondialdehyde as the biomarker evaluation of oxidative damage. The results showed that H_2_S could reverse the decrease of SOD, GSH-PX, and CAT and increase MDA induced by H_2_O_2_. In contrast, H_2_O_2_ suppressed the endogenous H_2_S production and H_2_S-producing enzymes CBS, CSE, and MPST, to further enhance oxidative stress. Recent studies have demonstrated that cell apoptosis induced by ROS could activate the MAPK pathway [34]. ERK, JNK, and p38 are three of the main components in the MAPK family [35]. Our results indicate that H_2_O_2_ treatment triggered the phosphorylation of ERK and p38. However, pre-treatment with H_2_S significantly altered H_2_O_2_-induced p-ERK and p-p38, but slightly altered p-JNK. Meanwhile, H_2_S activated the phosphorylation of AKT to promote cell proliferation when A549 cells became injured. These results provide evidence for the critical roles of H_2_S in ROS-induced apoptosis via the MAPK signaling pathway.

Mitochondria are the main sites of oxidative phosphorylation and ATP production [36]. H_2_O_2_ exposure causes the irreversible damage of mitochondria and loss of Δψ in cardiac fibroblasts [37]. Our previous study showed that a low concentration (less than 10 μM) of H_2_S facilitated electron transport and participated in the regulation of mitochondrial respiration in a bovine heart in vitro [38]. Therefore, in this study, we aimed to investigate whether H_2_S is involved in mitochondria stabilization in H_2_O_2_-induced lung injury. The results showed H_2_O_2_ decreased Δψ and inhibited the activities of metabolic enzymes, reducing energy production in A549 cells. A lack of energy due to oxidative damage further aggravated cell damage. However, H_2_S, which facilitated glucose utilization and increased ATP production, played a protective role in human lung epithelial cells. These findings indicate that H_2_S neutralized the damaging effects of H_2_O_2_ in cell metabolism and mitochondria producing energy.

ER stress can activate apoptotic signals to remove the damaged cells. Under physiological conditions, GRP78 is bound to the three ER stress sensors inositol requiring enzyme 1 (IRE1), protein kinase RNA-like ER kinase (PERK), and activating transcription factor 6 (ATF6), to form a stable complex [39]. Under ER stress conditions, calcium dyshomeostasis and misfolded proteins accumulate in the ER. GRP78 is released from the sensors and triggers an unfold protein response (UPR) [40]. The stress sensors are activated, and in the meantime, up-regulate GRP78 and CHOP expression [41]. Our data indicate that H_2_O_2_ obviously increased the expression of GRP78 and CHOP. Following ER stress, three signaling pathways: the IRE1 pathway, the PERK/eIF2α/ATF4 pathway, and the ATF6 pathway, were activated [42]. All these pathways are capable of altering the levels of Bcl-2 family members to elicit apoptosis [43]. Therefore, these three pathways were examined in response to H_2_O_2_ treatment. In the IRE1 pathway, H_2_O_2_ increased the phosphorylation of IRE1, but H_2_S pre-treatment changed p-IRE1 expression slightly. In the second pathway, H_2_O_2_ activated p-ERK, resulting in a large increase of phospho-eIF2α and ATF4. However, only a little change in p-eIF2α and ATF4 could be observed with H_2_S pre-treatment. For the last pathway, the results showed that H_2_S treatment significantly inhibited H_2_O_2_-induced AFT6 up-regulation. These findings indicated that the protective effect of H_2_S against H_2_O_2_-induced injury was closely related to ER stress via the ATF6 pathway.

ER stress and oxidative stress together have an impact on many diseases, including diabetes, cardiovascular disease, and cancer [44,45,46]. Overwhelming ROS production disrupts the redox equilibrium in the ER lumen, leading to excessive ER stress and cell apoptosis [47,48,49]. Meanwhile, ER stress facilitates ROS overproduction and thus activates the Ca^2+^/XO/ROS/mPTP pathway [50]. Therefore, combination therapies that suppress both ROS and ER stress might be a potential therapeutic agent to protect lung injury. Our results show that pre-treatment with H_2_S could counteract ROS and ER stress processes and might provide an effective way to regulate the tumor microenvironment.

## 4. Materials and Methods

### 4.1. Cell Culture

The human lung epithelial cell line A549 was obtained from the Cell Bank of the Chinese Academy of Sciences (Shanghai, China). Cells were cultured in RPMI-1640 medium (Solarbio Science & Technology, Beijing, China) with 10% fetal bovine serum (FBS) at 37 °C and 5% CO_2_. NaHS and H_2_O_2_ were purchased from Sigma-Aldrich (St. Louis, MO, USA) and the solution was prepared immediately before use. A549 cells treated with serum-free medium, 50 μM H_2_S, and 60 mM H_2_O_2_ were the control group, H_2_S group, and H_2_O_2_ group, respectively. A549 cells pre-treated with H_2_S (50 μM) for 2 h and then subjected to H_2_O_2_ (60 mM) for 10 h were the protection group (H_2_S+H_2_O_2_). A549 cells were rinsed with phosphate buffer saline (PBS) buffer three times after H_2_S pretreatment, before subjecting the cells to H_2_O_2_ challenge. After 12 h of treatment, cells were then used for the subsequent experiments.

### 4.2. Cell Viability and Colony Formation

1.0 × 10^4^ cells were plated in 96-well plates to assess cell viabilities. Different concentrations of NaHS (0, 10, 25, 50, 100, 200 μM) and H_2_O_2_ (0, 20, 40, 60, 80, 100, 200 mM) were added to the serum-free medium. After 12 h treatment, cells were assessed via an MTT assay and the optical density was measured at 490 nm by a multifunction microplate reader (Tecan Infinite, Mannedorf, Switzerland).

3 × 10^2^ A549 cells/well were seeded in 6-well plates for a colony formation assay. After two weeks, colonies were fixed in methanol, stained with 0.1% crystal violet, and photographed to count the number.

### 4.3. Analysis of Cell Apoptosis

The apoptosis assays were measured with a BBcellProbe™ Annexin V FITC/PI Apoptosis Detection Kit (BestBio, Shanghai, China). Briefly, A549 cells were collected in 400 μL binding buffer and incubated with 5 μL Annexin V and 10 μL PI for 10 min. A CytoFLEX flow cytometer (Beckman Coulter Life Sciences, Indianapolis, IN, USA) was used to measure apoptosis rates. Caspase 3 activity was detected by a caspase 3 activity assay kit (BestBio), according to the manufacturer’s instructions. Briefly, after being lysed on ice for 30 min, cellular proteins were incubated in reaction buffer with Ac-DEVD-pNA at 37 °C for 4 h. The 405 nm absorbance was measured via NanoDrop 2000 apparatus (Thermo Fisher Scientific, Waltham, MA, USA).

### 4.4. Quantitative Real-Time PCR (qRT-PCR)

RNA was isolated using a Total RNA Purification Kit (BioTeke Corporation, Beijing, China), as per the manufacturer’s instructions, and reverse transcribed into cDNA using a PrimeScript™ RT Reagent Kit with gDNA Eraser (TaKaRa, Bio, Kyoto, Japan). qRT-PCR was performed using SYBR Green PCR Mastermix (Solarbio Science & Technology) and DNA amplification was performed using an Applied Biosystems ABI 7500 thermal cycler (Thermo Fisher Scientific). The results were calculated using the 2^−ΔΔCt^ method. β-actin was the internal control. The primer sequences are listed in Table 1.

### 4.5. Western Blot

Cellular proteins were extracted in RIPA lysis buffer (BioTeke Corporation) on ice. An equal protein content of cell lysates was loaded onto sodium dodecyl sulfate-polyacrylamide gel electrophoresis, electrophoretically resolved, and then transferred onto polyvinylidene difluoride western blot membranes (Roche, Basel, Switzerland). The membranes were blocked for 3 h at 25 °C in 5% skim milk, and then incubated with specific primary and secondary antibodies. Immunoblots were detected using an ECL Western Blotting Substrate (Solarbio Science & Technology) and visualized using a Tanon 5200 digital imaging system (Tanon Science & Technology, Shanghai, China). Primary antibodies were caspase-3, cleaved-caspase-3, Bcl-2, Bax, GRP78, CHOP, IRE1, elF2α, ATF4, ATF6, p-38, p-p38, ERK, p-ERK, JNK, p-JNK, AKT and p-AKT (Wanleibio, Shenyang, China), β-actin, MPST, p-IRE1 (Bioss, Beijing, China), CBS, CSE (Omnimabs, Alhambra, CA, USA), and p-elF2α (Abbkine, Wuhan, China). Secondary antibodies (goat anti-rabbit IgG/HRP antibody, goat anti-mouse IgG/HRP antibody) were purchased from Bioss. Western blotting quantification results were evaluated with Image J software.

### 4.6. Measurement of H_2_S in Cell Culture Supernatants

H_2_S production was tested using a methylene blue assay as per the manufacturer’s instructions (Solarbio Science & Technology). Briefly, the test is based on the reaction between H_2_S and zinc acetate that forms zinc sulfide, which is then dissolved in N, N-dimethyl-p-phenylenediamine sulfate. Upon ammonium ferric sulfate addition, methylene blue forms were then quantified from the absorbance read using a UV-VIS spectrophotometer (UV-2700, Shimadzu, Kyoto, Japan).

### 4.7. Measurement of [Ca^2+^]

[Ca^2+^] measurement was performed according to the manufacturer’s instructions (Solarbio Science & Technology). Briefly, A549 cells were incubated in hanks balanced salt solution (1% FBS) with Fluo-3, AM at 37 °C for 40 min. Then, cells were washed with HEPES buffer saline three times and examined using IX73 fluorescence microscopy (Olympus, Kyoto, Japan).

### 4.8. Measurement of Mitochondrial Membrane Potential (Δψ)

Mitochondrial membrane potential was measured with a mitochondria-specific cationic dye JC-1 (BestBio), according to the manufacturer’s instructions. Briefly, A549 cells were incubated in dye buffer with 5 μL JC-1 for 15 min at 37 °C and observed using an LSM710 laser scanning confocal microscope (Carl Zeiss, Oberkochen, Germany).

### 4.9. Reactive Oxygen Species (ROS), Malondialdehyde (MDA), Superoxide Dismutase (SOD), Glutathione (GSH), and Catalase-Peroxidase (CAT) Assays

ROS was measured with 2′,7′-dichloroflurescein-diacetate (DCFH-DA, BestBio) and observed using an LSM710 laser scanning confocal microscope (Carl Zeiss). MDA content (Wanleibio), SOD activity, GSH concentration, and CAT activity (Solarbio Science & Technology) were measured following the manufacturer’s instructions.

### 4.10. Metabolic Assays

ATP production was detected using an ATP Bioluminescent Assay Kit (Nanjing Jiancheng Bioengineering Institute, Nanjing, China), according to the manufacturer’s instructions. Glucose consumption assay, lactic acid production, pyruvate, and lactate dehydrogenase (LDH) were performed with a Glucose Measurement Assay Kit (Rongsheng Biotech, Shanghai, China), Lactic Acid Assay Kit (Nanjing Jiancheng Bioengineering Institute), Pyruvate Assay Kit (Nanjing Jiancheng Bioengineering Institute), and LDH Assay Kit (Wanleibio), respectively, according to the manufacturer’s instructions.

### 4.11. Statistical Analysis

All results were expressed as means ± standard deviation (SD) from at least three independent experiments. Data between-group differences were evaluated by two-tailed *t*-tests. SPSS 16.0 (IBM Corporation, Chicago, IL, USA) and GraphPad Prism 6.0 (GraphPad Software, La Jolla, CA, USA) software were used to perform all statistical analyses. Only results with *p*-value < 0.05 were considered statistically significant.

## 5. Conclusions

In summary, our results demonstrated that H_2_S reduced ROS production and markedly inhibited apoptosis induced by H_2_O_2_, as well as maintained the structural and functional integrity of the mitochondria in A549 cells. Moreover, H_2_S attenuated [Ca^2+^] overload and ER stress induced by H_2_O_2_. These findings might provide an effective way to counteract ROS and ER stress processes in H_2_O_2_-induced lung injury.

## Figures and Tables

**Table 1:** Quantitative real-time PCR (qRT-PCR) primers used in the study.

Gene	Forward (5′-3′)	Reverse (5′-3′)
CBS	AATGGTGACGCTTGGGAA	TGAGGCGGATCTGTTTGA
CSE	AAGACGCCTCCTCACAAGGT	ATATTCAAAACCCGAGTGCTGG
MPST	GACCCCGCCTTCATCAAG	CATGTACCACTCCACCCA
Bax	TGGCAGCTGACATGTTTTCTGA	TCACCCAACCACCCTGGTCTT
Bcl-2	CAGTTGGGCAACAGAGAACCAT	AGCCCTTGTCCCCAATTTGGAA
β-actin	CTGGAACGGTGAAGGTGACA	AAGGGACTTCCTGTAACAATGCA

## Supplementary Materials

Supplementary materials can be found at https://www.mdpi.com/1422-0067/20/16/3975/s1.

## Abbreviations

H_2_S	hydrogen sulfide
H_2_O_2_	hydrogen peroxide
CBS	cystathionine-beta-synthase
CSE	cystathionine-gamma-lyase
MPST	3-mercapto-pyruvate sulfurtransferase
ER	endoplasmic reticulum
MAPK	mitogen-activated protein kinase
ROS	reactive oxygen species
NaHS	sodium hydrosulfide
IC_50_	half maximal inhibitory concentration
MTT	3-(4, 5-dimethylthiazol-2-yl)-2, 5-diphenyltetrazolium bromide
DCFH-DA	2′,7′-dichloroflurescein-diacetate
SOD	superoxide dismutase
GSH-PX	glutathione peroxidase
CAT	catalase
MDA	malondialdehyde
Δψ	mitochondrial membrane potential
ATP	adenosine triphosphate
LDH	lactate dehydrogenase
IRE1	inositol requiring enzyme 1
PERK	protein kinase RNA-like ER kinase
ATF6	activating transcription factor 6
UPR	unfold protein response
FBS	fetal bovine serum
qRT-PCR	quantitative real-time PCR
PBS	phosphate buffer saline
NSAIDS	nonsteroidal anti-inflammatory drugs
