# Supplementary material for: Hydrogen Sulfide Attenuates Hydrogen Peroxide-Induced Injury in Human Lung Epithelial A549 Cells
Source: Int J Mol Sci. 2019 Aug 15;20(16):3975. doi: 10.3390/ijms20163975 (PMC6720512; doi:10.3390/ijms20163975)
Supplement: Supplementary file 1 [file ijms-20-03975-s001.pdf]

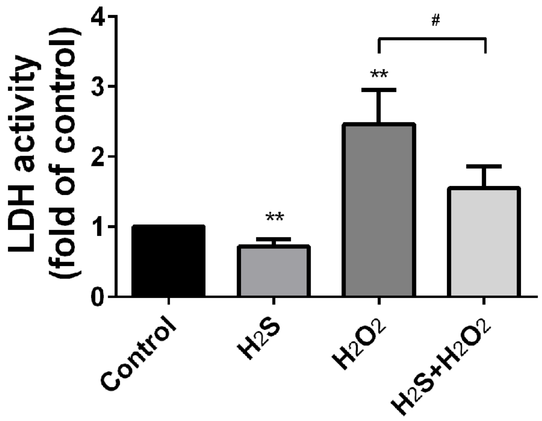

Figure S1. H_2O_2 increased LDH release in A549 cells.

The enzymatic activity of lactate dehydrogenase (LDH) in serum-free medium were measured. The experiments were repeated at least three times. The results are presented as the mean ± SD. (***P* < 0.01 vs. control group; #*P* < 0.05 H_2O_2 group vs. H_2S+H_2O_2 group).
